# Dynamics of nasopharyngeal tract phageome and association with disease severity and age of patients during three waves of COVID‐19

**DOI:** 10.1002/jmv.27998

**Published:** 2022-07-25

**Authors:** Carlo Ferravante, Berin S. Arslan‐Gatz, Federica Dell'Annunziata, Domenico Palumbo, Jessica Lamberti, Elena Alexandrova, Domenico Di Rosa, Oriana Strianese, Alessandro Giordano, Luigi Palo, Giorgio Giurato, Francesco A. Salzano, Massimiliano Galdiero, Alessandro Weisz, Gianluigi Franci, Francesca Rizzo, Veronica Folliero

**Affiliations:** ^1^ Department of Medicine, Surgery and Dentistry ‘Scuola Medica Salernitana', Laboratory of Molecular Medicine and Genomics University of Salerno Baronissi Italy; ^2^ Department of Experimental Medicine University of Campania “Luigi Vanvitelli” Naples Italy; ^3^ Genome Research Center for Health ‐ CRGS Campus of Medicine ‐ University of Salerno Baronissi Italy; ^4^ Department of Medicine, Surgery and Dentistry University of Salerno Baronissi Italy; ^5^ Medical Genomics Program, AOU ‘S. Giovanni di Dio e Ruggi d'Aragona’ University of Salerno Salerno Italy

**Keywords:** bacteriophages, phageome, SARS‐CoV‐2, taxonomic analysis

## Abstract

In December 2019, several patients were hospitalized and diagnosed with severe acute respiratory syndrome coronavirus 2 (SARS‐CoV‐2) infection, which subsequently led to a global pandemic. To date, there are no studies evaluating the relationship between the respiratory phageome and the SARS‐CoV‐2 infection. The current study investigated the phageome profiles in the nasopharyngeal swabs collected from 55 patients during the three different waves of coronavirus disease 2019 (COVID‐19) in the Campania Region (Southern Italy). Data obtained from the taxonomic profiling show that phage families belonging to the order *Caudovirales* have a high abundance in the patient samples. Moreover, the severity of the COVID‐19 infection seems to be correlated with the phage abundance.

## 1 INTRODUCTION

Based on the data provided by the World Health Organization, coronavirus disease (COVID‐19) has affected more than 515 million people around the world.^1^ While most patients showed heterogeneous mild or no symptoms, about 14% developed severe clinical signs and more than 6.2 million deaths have been recorded up to date.

The microbiota has a crucial role in human health and its impairment has been noted in several infectious disorders.^2^ Alteration in bacterial microbiota was observed among patients with a wide range of viral respiratory infections.^3^ In a study by Edouard et al., patients infected with influenza A and B viruses, rhinovirus, metapneumovirus, and respiratory syncytial virus showed a reduction of anaerobic bacteria and an invasion of pathogenic bacteria, including *Staphylococcus aureus, Haemophilus influenzae, Streptococcus pneumoniae, Moraxella catarrhalis, Dolosigranulum pigrum*, and *Corynebacterium pseudodiphtheriticum*.^4^ Evidence revealed that compositional and functional shifts in the microbiota affect susceptibility and the disease.^5^ Piters et al. demonstrated that the severity of respiratory syncytial virus bronchiolitis was positively associated with the high abundance of *Streptococcus* and *H. influenzae* and negatively correlated with the presence of *S. aureus* in the nasal tract.^6^ Similar observations were published by Stewart et al., who reported strong interrelationships between *Streptococcus*‐dominant nasal microbiome and symptoms of viral bronchiolitis.^7^ Our past studies revealed that COVID‐19 patients with severe outcomes had a nasopharyngeal district with reduced Proteobacteria and increased *Rothia mucilaginosa, Streptococcus oralis*, and bacterial species belonging to the genera *Prevotella* and *Veillonella*.^8^ One of the most underestimated aspects of the microbiome is bacteriophages (phages). Phages are viruses that infect and replicate only in bacterial cells. Like all viruses, phages are species‐specific, infecting a single bacterial species or a subpopulation thereof. By the International Committee on Taxonomy of Viruses (ICTV), phages are classified according to their morphology, nucleic acid type, host range, replication cycle, and sequence similarity.^9^ *Siphoviridae, Inoviridae, Myoviridae*, and *Podoviridae* are among the well‐known phage families.^10^ A certain amount of evidence reports the essential role of phages in the composition of bacterial communities in different environments.^11^ Moreno‐Gallego et al. showed that the nature of the microbiome depended on the phage population, whose composition is closely related to disease.^12^ Indeed, Bacteroides phages and phages belonging to the *Siphoviridae, Myoviridae*, and *Podoviridae* families were known to be abundant in fecal samples of patients with Crohn's disease, explaining the high Bacteroides burden in the intestines of these patients.^13^ Given the importance of the microbiota in the progression of diseases and the close relationship of phages with the composition of the bacterial community, the current study investigated the nasopharyngeal phageome in COVID‐19 patients with different disease severity during three different pandemic waves. To our knowledge, no studies evaluating this aspect of the disease are reported to date in the main databases. Moreover, the nasopharyngeal phageome of COVID‐19 patients could potentially be used as a noninvasive diagnostic tool for assessing the progression of the disease.

## 2 MATERIALS AND METHODS

### 2.1 COVID19 patients

Fifty‐five SARS‐CoV‐2‐positive patients from the Campania region (Southern Italy), were included in this study. For symptomatic COVID‐19 patients, nasopharyngeal swab specimens were collected soon after the symptom onset. For asymptomatic COVID‐19 patients, swabs were performed 5–7 days after close contact with a COVID‐19 patient. Viral infection was confirmed by positive molecular tests. Nasopharyngeal swabs samples were collected during the three main SARS‐CoV‐2 outbreaks in Italy, and they were divided into three main groups: the first group (collection date: March–May 2020); the second group (collection date: September–November 2020); and the third group (collection date: January–February 2021). In total, 25 samples belonged to the first, as well as the second groups and 5 to the third. Thirty‐one percent of patients were female (*n* = 17), and 65% were male (*n* = 36), with a median age of 59 years, ranging from 8 to 91 years. The study was approved by the Ethics Committee of “Campania Sud” (approval code: 206/2021) and was conducted according to the Declaration of Helsinki. Samples were also divided based on the severity of symptoms and were clustered into nonsevere (*n* = 39), moderate (*n* = 6), and severe (*n* = 10) as described in Ferravante et al.^14^ The clinical characteristics of the patients are summarized in Table 1. An important limitation of the study is the lack of healthy controls and multiple samplings for each COVID19 patient over time. The reference ethics committee and the proposed study involved the collection of a single nasopharyngeal sample per patient. Understanding the differences in nasopharyngeal phageome between COVID‐19 patients with different symptoms and over time could be of great importance for clinical management.

**Table 1:** Clinical characteristics of 55 patients enrolled in the study

	Mar–May 2020 (*n* = 25)	Sep–Nov 2020 (*n* = 25)	Jan–Feb 2021 (*n* = 5)
Age			
8–40	6	7	–
41–59	3	10	–
60–69	5	4	4
>70	8	4	1
Unknown	3	–	–
Gender			
Male	15	17	4
Female	8	8	1
Unknown	2	–	–
Disease severity			
Nonsevere	18	21	–
Moderate	2	–	4
Severe	5	4	1

### 2.2 Library preparation, sequencing, and bioinformatics analysis

To extract total RNA, the 55 nasopharyngeal swabs were processed using ELITeInGenius fully automated system (ELITechGroup) and ELITeInGenius SP RNA cartridge (ELITechGroup). RNAs extracted were then retro‐transcribed using SensiFAST™ cDNA Synthesis Kit (meridian BIOSCIENCE). Real‐time polymerase chain reaction (RT‐PCR), was used to measure the viral load of each sample, by targeting the Sars‐CoV‐2 viral nucleoprotein with the following primers:

- Forward: GGGGAACTTCTCCTGCTAGAAT
- Reverse: CAGACATTTTGCTCTCAA

The nucleic acid concentration was assessed using Qubit RNA HS Assay Kit (Thermo Fisher Scientific) and libraries were generated using 100 ng of RNA, with TruSeq Stranded Total RNA Kit (Illumina) according to the manufacturer's instructions. In the first step, ribosomal RNA was depleted, then the RNA was fragmented, and the first‐strand complementary DNA filament was synthesized. For the synthesis of the second strand, dUTPs instead of dTTPs were used to extinguish the amplification of the second strand during the PCR amplification step and consequently to the adenylation of double‐strand DNA (dsDNA) fragments, indexed adapters were ligated and DNA fragments containing adapter molecules were enriched by 15 PCR cycles. With Qubit dsDNA HS Assay Kit (Thermo Fisher Scientific), the final library concentration was measured, and library size was checked by Agilent 4200 Tapestation System (Agilent), showing an average size of 400 bp. The libraries were then sequenced on the NextSeq 500 (Illumina) in 2 × 75 paired‐end mode at a final concentration of 1.7 pMol or on NovaSeq 6000 (Illumina) in 2 × 100 bp paired‐end mode at a final concentration of 250 pMol. Raw fastq files were imported in the HOME‐BIO pipeline.^15^ The “Quality control” module was set to remove low‐quality reads and host‐related sequences, filtering out reads that mapped on the human reference genome (GRCh38.p13 release 37). Phage taxonomy profiling was obtained by querying RefSeq complete viral genomes/proteins database. Classification reports were then processed in R software (version 3.6.3) and normalized in reads per million (RPM) values (RPM mapped on the viral database). The raw read number obtained with the HOME‐BIO pipeline was normalized in million mapped reads on the viral database. This normalization criterion was chosen to avoid problems related to different sequencing depths and to highlight all the small differences in terms of read abundance, as described in Di Gaspero et al.^16^ Phage families identified by less than three reads in at least 50% of the analyzed samples were filtered out from the analysis. Then, the differential distribution of phage families was computed as a ratio between the mean values of RPM in the groups analyzed. Statistical significance was computed by applying a *T*‐test followed by Bonferroni correction. Only comparisons with a *p* value ≤ 0.05 were considered significant, as described in Giugliano et al.^8^

## 3 RESULTS AND DISCUSSION

**Figure 1:**
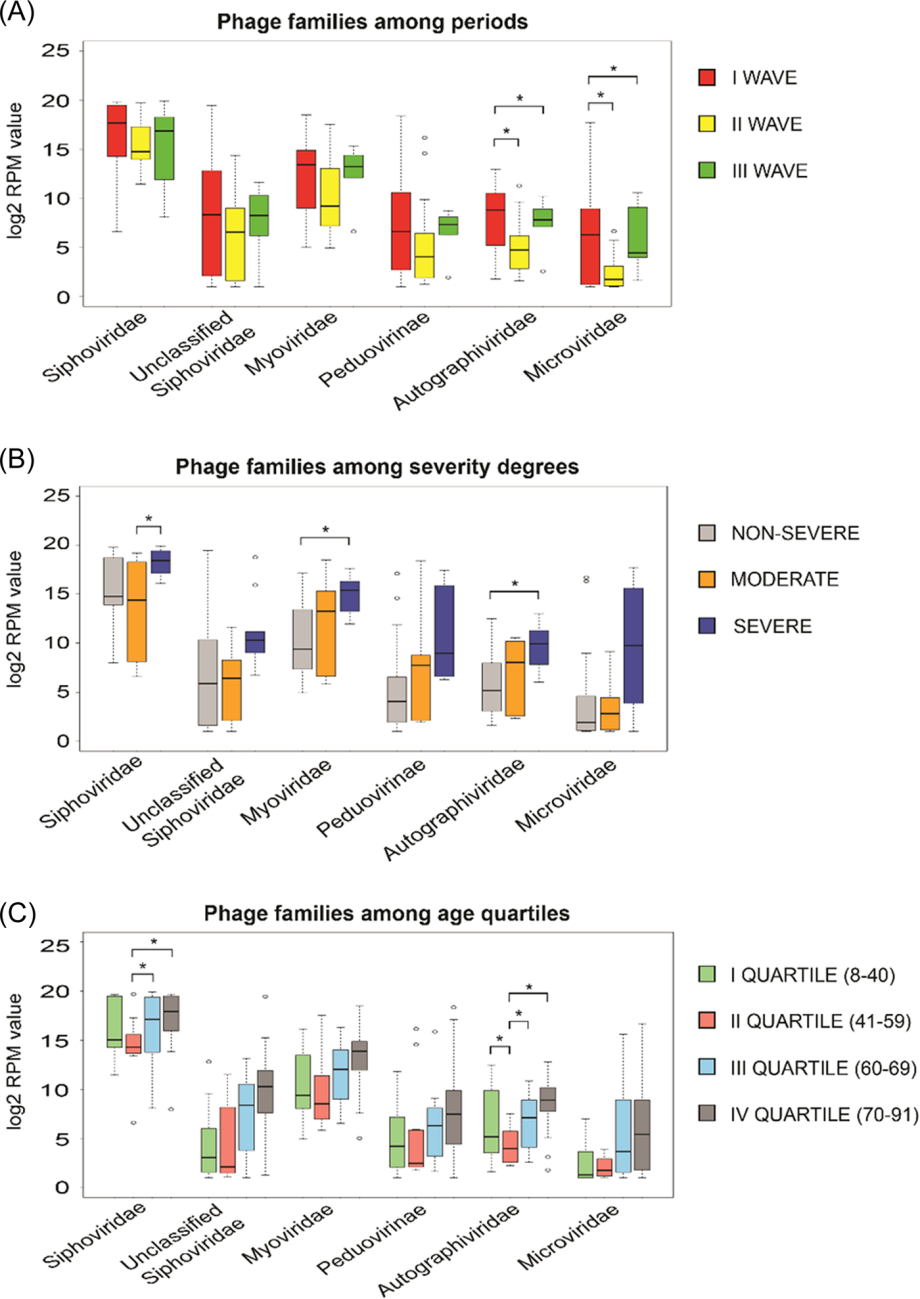
Distribution of RPM values related to detected phage families among the three time periods (A), disease severity degrees (B) and age quartiles (C). The significant comparisons between analyzed groups, associated with a *p*‐value ≤ 0.05, are indicated with an asterisk (*).

The sequencing of 55 RNA samples from nasopharyngeal swabs of SARS‐CoV‐2 positive patients generated more than 6.03 billion reads, with an average of 109 809 145 reads per sample (range 52 888 010–228 793 156 reads). The HOME‐BIO's “Quality Control module” filtered out about 1.3 million reads per sample associated with low‐quality and adapter sequences. Furthermore, about 54% of the remained reads per sample were mapped on the human reference genome and were excluded from the downstream analysis, resulting in a total of 43 184 475 reads per sample (range 4 610 220–147 612 628 reads) that passed the “Quality Control module.” On the entire data set more than 358 million reads were mapped on a viral database, corresponding to 18% of analyzed reads per sample (mean: 6 510 568; range: 1718–40 900 812). Only sequences that matched on phages have been considered in this paper as virus and bacteria presence have been already described respectively in Giugliano et al.^8^ and Ferravante et al.^14^ A total of six phage families that passed filters (phage entities were retained if they had a minimum of three reads associated in at least 50% of samples analyzed), were considered as detected in the 55 nasopharyngeal swabs taken from the patients infected with SARS‐CoV‐2. The RPM mean value of the six phage families detected, related to the three waves, different degrees of severity as well as the age quartile groups are reported in Table 2. As predicted, *Siphoviridae* appears to be the most abundant of all phage families detected with an average of 266 428.02 RPM value on the entire data set, which is more than nine times higher than the second most abundant *Myoviridae* family (associated with 27 386.35 RPM mean value on the entire data set). Focusing on the three different pandemic waves, *Siphoviridae* remains to be the most abundant phage family, followed by *Myoviridae* in the II and III waves of COVID‐19. However, in the I pandemic period, the second most abundant phage family is the *unclassified Siphoviridae*. Overall, the phage abundance has the highest RPM values in the I wave, and the RPM values decreased in waves II and/or III (Figure 1A). The reasons behind this decrease may be explained by the restrictions such as wearing masks, reducing human contact, maintaining distance, and even strict curfews applied around the world during the pandemic. While *Microviridae* belongs to the order Petitvirales, all others *Siphoviridae, unclassified Siphoviridae, Myoviridae, Peduovirinae* (subfamily), and *Autographiviridae* belong to the order *Caudovirales*. All detected phage families are dsDNA viruses, apart from *Microviridae*, which are ssDNA viruses. The phages found in the presented study are highly similar to the natural phage communities identified in the respiratory tract of the human body.^17^, ^18^, ^19^, ^20^ The difference in phage abundance is clearly visible when comparing the first wave with the second and third waves. In the first wave, overall mean phage abundance was higher for all three families than those in the II and III waves. The highest difference in RPM values was found in the *Peduovirinae* subfamily during the I and III waves, associated with an RPM mean values of 28 745.07 and 187.22, respectively. *Autographiviridae* and *Microviridae*, instead, were more abundant in wave I than in wave II. In detail, the *Autographiviridae* family was associated with an RPM mean value of 1,438.58 and 192.85 in the I and II wave, respectively. A similar trend was shown by the *Microviridae* family which was represented by 23,196.82 and 8.92 RPM mean values in the I and II period, respectively. Similarly, *Peduovirinae, Autographiviridae*, and *Microviridae* showed different RPM mean values in the first wave compared to the third one. In the same way, *Autographiviridae*, and *Microviridae* families resulted more abundant in the first period (associated with 1438.57 and 23 196.82 RPM mean values respectively), than in the third wave, which displayed an RPM mean value of 399.69 and 416.04 for *Autographiviridae*, and *Microviridae*, respectively. *Siphoviridae, Myoviridae*, and unclassified *Siphoviridae* did not show any significant difference in abundance among all three waves. More interestingly, we noticed a possible association between the phage abundance and the disease severity. Overall, all phage families detected showed higher RPM values in patient samples with severe symptoms in comparison to those classified as nonsevere. *Siphoviridae*, showed 429 879.71 RPM mean value in the severe cases. This phage family showed significant differences (*T*‐test *p* value = 0.04) when compared to patients with moderate symptoms (associated with RPM mean value of 172 107.89). The comparison between severe and nonsevere COVID 19 patients highlighted an interesting scenario. *Myoviridiae* and *Autographiviridae* were less abundant in patients without symptoms (RPM mean value of 10 626.78 and 444.11 respectively) than in the severe group characterized by RPM mean value of 66 317.31 and 2220.94 for *Myoviridiae* and *Autographiviridae*, respectively. While the difference between severe and nonsevere COVID‐19 patients according to RPM readings is significant in the *Autographiviridae* family, the family has the least number of RPM reads detected compared to other families (Figure 1B). Although *Autographiviridae* is not a dominant member of the human phageome, this phage family has clinical importance, in fact, has been associated quite often with pathogenic bacteria causing serious illnesses, especially in the hospital environment.^21^, ^22^ *Siphoviridae* has the highest number of reads in all clinical outcomes. The phage abundance drastically reduces from the severe cases to the nonsevere cases. In a recent study involving COVID‐19 patients, *Myoviridae, Siphoviridae, Microviridae, Podoviridae*, and crass‐like phages were detected in the virome of fecal DNA of patients.^23^ The results from the study correlate with our findings. In addition, the study reported that the virome composition in the gut changed significantly in the COVID‐19 patients when compared with healthy controls and the significant changes might be related to the changes in the bacteriome composition. A study conducted in China on fecal samples collected from COVID‐19 patients showed that some phages including *Microviridae* were remarkably more abundant in COVID‐19 patients than in healthy subjects.^24^ Despite the lack of comparison to healthy controls, in our study, the abundance of the *Myoviridae* family was higher in the patients with a severe infection in comparison to the non‐symptomatic patients. In addition to being enriched during the COVID‐19 infection, the *Myoviridae* phage family was found to be in a significant correlation with other diseases such as inflammatory bowel disease, while *Siphoviridae* were found to be associated with T2D.^25^, ^26^ Furthermore, we looked for a possible correlation with the patient's age, for this purpose samples were stratified into four groups based on age quartile distribution. *Siphoviridiae* and *Myoviridae* families were more abundant in the patients of the III and IV age quartiles. All the detected phage families seem to have more similar RPM reads for the I and II age quartiles than those of the other age groups (Figure 1C). Features like genetics, breastfeeding, aging, diseases, medication, and geography are known to alter the development of the human virome.^20^ The phages are thought to be introduced to the body with prophage induction from early bacteria colonies.^27^ According to recent research studies, early colonizers in the human body were mostly phages from *Siphoviridae, Podoviridae*, and *Myoviridae* families.^20^ The analysis of four different age groups has shown that the abundance of the *Microviridae* family increases with age, but the differences measured in terms of RPM mean value are not supported by a significant *p* value. Our results correlate with the two recent studies on the nature of human virome from early life and its further development in the body.^28^ When compared among different age groups, only two phage families showed significant differences according to *T*‐test results: *Siphoviridae* and *Autographiviridae*. In detail, *Autographiviridae* was less abundant in the 41–59 years old patients (RPM mean value = 38.41) than in all other groups (0–40 year RPM mean value = 840.67; 60–69‐year RPM mean value = 413.77, >70‐year RPM mean value = 1292.56). Similarly, a decreased level of reads related to the *Siphoviridae* family was detected in patients belonging to the II age group (age range: 41–59 years, RPM mean value = 103 121.49) when compared with those of the III and IV (associated with 326 859.45 and 358 966.02 RPM mean value, respectively).

**Table 2:** Reads associated with each family detected among waves, degrees of severity, and age groups

		Phage families					
		*Siphoviridae*	*Unclassified Siphoviridae*	*Myoviridae*	*Peduovirinae*	*Autographiviridae*	*Microviridae*
WAVES	I	332 155.73	53 871.06	42 950.89	28 745.07	1438.58	23 196.82
	II	197 346.73	1247.60	14 275.20	3996.05	192.85	8.92
	III	283 195.91	950.69	15 119.46	187.22	399.69	416.04
SEVERITY	Non‐severe	239 028.12	22 252.13	10 626.78	4427.21	444.11	4819.95
	Moderate	172 107.89	600.97	71 438.63	56 010.57	542.39	96.59
	Severe	429 879.71	51 128.08	66 317.31	31 073.96	2220.95	39 366.65
AGE QUARTILE	I	286 672.37	645.85	13 999.70	474.40	840.67	23.49
	II	103 121.49	318.05	17 470.69	7503.70	38.41	3.35
	III	326 859.45	1416.88	12 762.49	4698.67	413.77	4018.31
	IV	358 966.02	59 940.92	51 546.85	36 772.71	1292.56	16 403.24

*Note*: Values are reported as a mean of normalized RPM in the analyzed group.

## 4 CONCLUSION

In the presented study, an analysis of nasopharyngeal swabs was performed to taxonomically classify the bacteriophage population in the patients from the Campania region infected with SARS‐CoV‐2. The total RNA content of the samples was deeply investigated using HOME‐BIO,^15^ a recently developed pipeline, which provided an exhaustive analysis of phage abundances during 3 main periods of COVID‐19 infection and related them to a different degree of severity of the illness. On the whole, we have shown that disease severity and age can be associated closely with the phage abundance in COVID‐19 patients. The taxonomic profiling analysis revealed that phage families belonging to the order *Caudovirales*, more prominent *with the families Siphoviridae* and *Myoviridae*, have a high abundance in the patient samples. Considering the abundance of bacteriophages in the human body, as well as in the respiratory tract, and their relation to the bacterial communities; the effect of phages on diseases like COVID‐19 has crucial clinical importance. For this reason, studying the changes in the human phageome will contribute significantly to better understanding the impact of phages on health and diseases. Importantly, we believe that the phageome of COVID‐19 patients has the potential to be used as a diagnostic tool to monitor the changes in the disease development.

## AUTHOR CONTRIBUTION

Study concept and design: Alessandro Weisz, Francesca Rizzo, Veronica Folliero, and Gianluigi Franci. Sample preparation and sequencing: Elena Alexandrova, Jessica Lamberti, Luigi Palo, and Domenico Di Rosa. Bioinformatics analysis: Carlo Ferravante, Domenico Palumbo, Alessandro Giordano, and Giorgio Giurato. Statistical analysis and interpretation of the data: Berin S. Arslan‐Gatz, Veronica Folliero, Gianluigi Franci, Alessandro Weisz, and Francesca Rizzo. Writing of the manuscript: Carlo Ferravante, Berin S. Arslan‐Gatz, Veronica Folliero, Federica Dell'Annunziata. Writing—review and editing: Alessandro Weisz, Oriana Strianese, Gianluigi Franci, Francesca Rizzo, Francesco A. Salzano, and Massimiliano Galdiero.

## CONFLICT OF INTEREST

The authors declare no conflict of interest.

## Data Availability

The data that support the findings described here are available from the corresponding authors upon request.
